# A problem formulation framework for the application of in silico toxicology methods in chemical risk assessment

**DOI:** 10.1007/s00204-024-03721-6

**Published:** 2024-03-30

**Authors:** Jerry Achar, Mark T. D. Cronin, James W. Firman, Gunilla Öberg

**Affiliations:** 1 Institute for Resources Environment, and Sustainability, The University of British Columbia, 2202 Main Mall, Vancouver, BC V6T 1Z4 Canada; 2 School of Pharmacy and Biomolecular Sciences, Liverpool John Moores University, Byrom Street, Liverpool, L3 3AF UK

**Keywords:** In silico methods, Risk assessment, Problem formulation, Uncertainty

## Abstract

The first step in the hazard or risk assessment of chemicals should be to formulate the problem through a systematic and iterative process aimed at identifying and defining factors critical to the assessment. However, no general agreement exists on what components an in silico toxicology problem formulation (PF) should include. The present work aims to develop a PF framework relevant to the application of in silico models for chemical toxicity prediction. We modified and applied a PF framework from the general risk assessment literature to peer reviewed papers describing PFs associated with in silico toxicology models. Important gaps between the general risk assessment literature and the analyzed PF literature associated with in silico toxicology methods were identified. While the former emphasizes the need for PFs to address higher-level conceptual questions, the latter does not. There is also little consistency in the latter regarding the PF components addressed, reinforcing the need for a PF framework that enable users of in silico toxicology models to answer the central conceptual questions aimed at defining components critical to the model application. Using the developed framework, we highlight potential areas of uncertainty manifestation in in silico toxicology PF in instances where particular components are missing or implicitly described. The framework represents the next step in standardizing in silico toxicology PF component. The framework can also be used to improve the understanding of how uncertainty is apparent in an in silico toxicology PF, thus facilitating ways to address uncertainty.

**Supplementary Information:**

The online version contains supplementary material available at 10.1007/s00204-024-03721-6.

## Introduction

In silico toxicology models (e.g., quantitative structure–activity relationships (QSAR) and read-across) form part of a broader collection of non-animal testing approaches that aim to reduce the reliance on animal testing and improve the prediction of potential harm caused by chemicals (Patlewicz et al. 2013; Pradeep et al. 2020; Schultz et al. 2015; Wang et al. 2012). The basic assumption is that the activity of a substance is relatable, either qualitatively or quantitively, to its molecular structure. As such, compounds displaying similarity in terms of chemical features or properties will additionally be anticipated to exhibit likeness in toxic profiles (Cronin and Madden 2010; Cronin et al. 2013; Enoch 2010). In silico approaches are particularly important in the risk assessment of chemicals such as cosmetic ingredients within jurisdictions including the European Union (EU) [Regulation (EC) No. 1223/2009) (European Commission 2016)], Canada (Bill S-5, Clause 16.1 (Government of Canada 2023)), and the United States (TSCA 4(h)(2)(C) (US EPA 2018)), where animal testing is prohibited or under consideration for prohibition for such use. However, the uncertainty associated with the model predictions is often referred to as one reason for low confidence and regulatory acceptance of in silico model predictions. Uncertainty in such predictions may arise from, amongst other factors, concerns regarding the quality and appropriateness of the training data, the extent of chemical applicability domain, and the interpretability of the relationship between input features and output (Blackburn and Stuard 2014; Parish et al. 2020; Schultz et al. 2019). Accordingly, it is often advised that these methods be applied alongside other non-animal testing approaches (e.g., in vitro tests) to complement the weight of evidence generated by them (Gautier et al. 2020). It has been postulated that formulation of frameworks and guidelines making it possible to systematically and transparently identify the many possible sources of these uncertainties would, in turn, increase the confidence in the utility of in silico toxicology methods (Alexander-White et al. 2022; Patlewicz et al. 2015). Several initiatives are thus underway to support the development of such frameworks to improve the consistency, quality, rigour, and reliability of chemical risk assessment procedures. For example, the 2017 European Chemicals Agency’s Read-Across Assessment Framework (RAAF), which aims to facilitate the development of a consistent, structured, and transparent read-across review process (European Chemicals Agency 2017). The US Environmental Protection Agency (US EPA) also recently launched a plan to develop a scientific confidence framework to evaluate the quality, reliability, and relevance of non-animal testing approaches, including in silico methods for regulatory chemical risk assessment (US EPA 2021). While considerable attention has been paid to identifying sources of uncertainty relatable to the various phases of model construction and application (Ball et al. 2014; Blackburn and Stuard 2014; Cronin et al. 2019a, b; Escher et al. 2019; Johnson et al. 2022; Patlewicz et al. 2015; Pestana et al. 2021; Pham et al. 2019; Rathman et al. 2018; Schultz et al. 2015, 2019; Viceconti et al. 2021), comparatively little focus has been dedicated towards addressing uncertainty liable to arise during problem formulation (PF).

Ideally, the first step in the hazard or risk assessment of chemicals is to formulate the problem through a systematic and iterative process aimed at identifying and defining factors critical to the assessment (Devos et al. 2019; Embry et al. 2014). When it comes to assessing the potential for harm posed by chemicals, it is argued that the PF ought, for example, to incorporate stages covering the characterization of the scope and context of the assessment, the identification of research needs, the development of a conceptual model and the formulation of a hypothesis (Devos et al. 2019; Embry et al. 2014; Paoli et al. 2022; Raybould 2006; Sauve-Ciencewicki et al. 2019; Solomon et al. 2016; Tepfer et al. 2013; Wolt et al. 2010). The US EPA introduced the concept of PF to risk assessment in 1998, applying it within an ecotoxicological setting (US EPA 1998). Its importance within the field is increasingly emphasized and endorsed, not only by individual scientists (Parish et al. 2020; Raybould 2006; Sauve-Ciencewicki et al. 2019; Solomon et al. 2016; Tepfer et al. 2013; Wolt et al. 2010) but also by regulatory agencies, research organizations and international bodies. Examples of these include the US EPA and the European Food Safety Authority (EFSA) (Devos et al. 2019; US EPA 2016), the National Research Council of the National Academy of Sciences (NRC) (Meek et al. 2013), and the Organization for Economic Cooperation and Development (OECD) (OECD 2019). Several recent studies applying in silico methods, or discussing the methods more generally, have also emphasized the need to include PF as the first step in the development and application of the methods for chemical risk assessment (Alexander-White et al. 2022; Escher et al. 2019; Ouedraogo et al. 2022; Parish et al. 2020; Raybould 2006; Reynolds et al. 2021). Essentially, in silico toxicology methods such as QSAR and read-across differ from other non-animal testing approaches (e.g., in vitro tests)—for example, with regards to the complex mathematical tools and algorithms, big data, and model parameters used. This suggests the need for producers of model output (e.g., model users) to have access to a framework that allows them to define a context specific PF that covers the complexities unique to in silico methods. There is, however, no general agreement on what a PF for studies applying in silico toxicology methods should include to strengthen the utility of such a PF and the related method itself. The lack of agreement makes it difficult to identify potential weaknesses in a PF, such as when particular components are missing. A missing central component may lead to an inadequately formulated problem, which in turn may result in an inadequate specification of risk concerns or provide insufficient clarity regarding the applicability domain of a model or scope of model predictive output. Accordingly, agreement on what a PF for an in silico toxicology method should include has the capacity to reduce the associated uncertainty and thus enhancing its utility.

Through providing an explicit and systematic evaluation of appropriate concepts, this study aims to contribute to the development of a PF framework relevant to the application of in silico methods for chemical toxicity prediction. This was performed by sourcing and examining a series of recent publications within which PF is considered in the predictive toxicology context. Components integral to the PFs in these studies—such as the endpoints addressed, the pathways of chemical exposure covered and the scope of model use intended—were analyzed in light of PF processes, as described in broader risk assessment literature [e.g., (Devos et al. 2019; Nickson 2008; Paoli et al. 2022; Raybould 2006; Sauve-Ciencewicki et al. 2019; Solomon et al. 2016; Wolt et al. 2010; World Health Organization/International Programme on Chemical Safety 2018)]. Subsequently, these components were grouped into appropriate component categories. Once complete, we set out to answer the questions: what PF components should be considered when developing PF for in silico toxicology methods, and how might exclusion or implicit description of the PF components introduce uncertainty into the method’s PF?

## Materials and methods

### Identifying PF components in the general risk assessment literature

We identified PF components described in the general risk assessment literature. Relevant documents, i.e., those describing a range of higher-level PF conceptual components potentially relevant to the application of in silico toxicology methods, were identified through a search in the Web of Science using two broad keywords and Boolean: (topic) “problem formulation” AND “risk assessment”, identifying 221 papers. The titles and abstracts of the identified papers we skimmed to identify 12 relevant pee-reviewed papers. Three relevant grey literature sources (i.e., the OECD (OECD 2019), the USEPA (US EPA 2016), and the World Health Organization/International Programme on Chemical Safety (World Health Organization/International Programme on Chemical Safety 2018)) were also identified after skimming the reference list of the 12 papers, rendering a total of 15 papers (see Table S1). A content analysis (Tracy 2018) of the 15 documents was carried to identify higher-level PF conceptual PF components discussed in them.

### Formulating a general PF framework

One of the 15 papers (Sauve-Ciencewicki et al. 2019) explores and formalizes PF concepts. We decided to use these general concepts as representations of higher-level PF components, as we found that these concepts cover a considerable amount of the component information mentioned in the other 14 publications. For example, problem framing, as described by Sauve-Ciencewicki et al. (2019), includes defining whether an assessment is intended for hazard or risk analysis (Felter et al. 2021), what qualifies as harm (Raybould 2006; Viceconti et al. 2021), potential chemical exposure scenario (Baltazar et al. 2020; Escher et al. 2019), and scientific questions to be addressed (Paoli et al. 2022). Guided by the discussions in the other 14 publications in Table S1, we expanded the framework by Sauve-Ciencewicki et al. (2019] from two higher-level components—problem framing and problem exploration—to four higher-level components—problem framing, problem exploration, conceptual model, and hypothesis formulation, as each of these need to be considered as distinct phases of PF (Devos et al. 2019; OECD 2019; Solomon et al. 2016; US EPA 2016; Wolt et al. 2010).

### Applying the PF framework to in silico toxicology methods

To apply the PF framework outlined in the Section "Identifying PF components in the general risk assessment literature" to in silico toxicology methods, we identified publications on in silico toxicology methods that describe PF as part of the method applications. This was done through a literature search in the Web of Science using the following broad keywords and Booleans: (topic) "in silico*" OR new approach methodologies OR NAMs OR non-animal testing OR alternative to animal testing OR read-across OR QSAR OR Comput Toxicol AND (all fields) "problem formulation." Out of the 112 publications identified, 13 papers (see Table S2) were selected based on the following criteria: peer-reviewed, relating to in silico methods and describing PF associated with in silico toxicology methods. These papers were analyzed to identify PF components described in them, whereafter the higher-level components identified under Section "Identifying PF components in the general risk assessment literature". were discussed in light of these components. In so doing, we acknowledge that it is possible that the procedure applied here might have led to some components in the in silico toxicology methods literature not being captured. However, since the conceptual breadth of our framework was not based on all possible components present in the literature, we considered the components identified in this section to be sufficient for our discussion.

## Results and discussion

### PFs for in silico toxicology methods

In chemical risk assessment, the problem at hand is to decide whether the potential harm posed by a chemical within a given scenario is sufficient to warrant concern (Wolt et al. 2010). To improve clarity and reduce uncertainty as to whether or not adverse effects could realistically arise from exposure to the target substance, the broader PF literature emphasizes that three higher-level, conceptual and context-specific questions must be addressed:

1. What must happen for harm to occur?
2. What is the likelihood of harm?
3. Is there a reasonable pathway to harm?

Hypotheses drawn from answers to these questions then form a basis to identify specific PF components relevant to the assessment (Sauve-Ciencewicki et al. 2019). In other words, to define the problem, it is necessary that the problem is first framed and explored (Sauve-Ciencewicki et al. 2019)—then that the associated research needs are identified (OECD 2019), and then subsequently that a conceptual model is developed detailing the nature of the variables (i.e., descriptors and endpoint) incorporated (Solomon et al. 2016). Subsequently, this may then guide the formulation of a specific causal pathway towards toxicity (Devos et al. 2019). When analyzing the 13 papers identified under Section “Formulating a general PF framework” (Table S2), we found that these aspects are not included in these papers, even though they specifically address PF in relation to in silico toxicology methods. This clearly reinforces the need for a PF framework for in silico toxicological methods that enables users of in silico models to answer the three central questions outlined above and identify context-specific PF components.

**Fig. 1:**
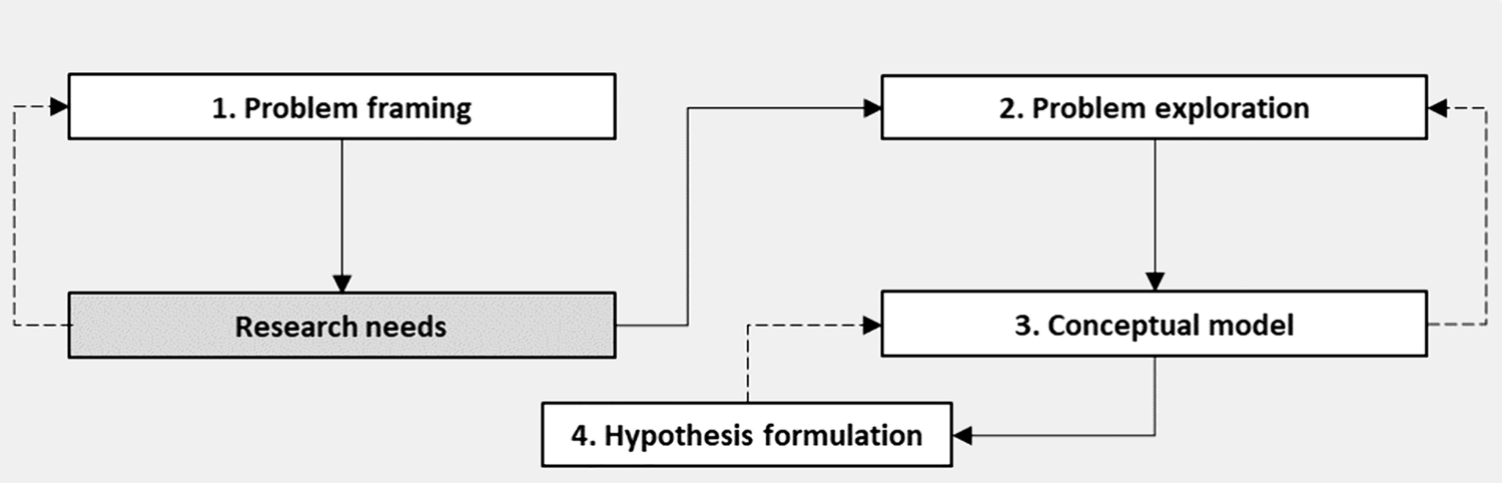
The problem formulation framework utilized in the present paper, outlining the iterative process from problem framing to hypothesis formulation [modified from Sauve-Ciencewicki et al. (2019)]

Similar to Sauve-Ciencewicki et al. (2019) and others (Devos et al. 2019; Embry et al. 2014; Raybould 2006; Solomon et al. 2016; Wolt et al. 2010), we interpret PF as an iterative process that begins with problem framing and ends with hypothesis formulation (Fig. 1). Between problem framing and hypothesis formulation, it proceeds through phases including the evaluation of available data and information, the determination of a preliminary understanding of potential harm, the identification of research needs, and the development of a conceptual model. The five key stages (problem framing, problem exploration, research needs, conceptual model development, and formulation of hypothesis), alongside the connectivity present between them, are illustrated in Fig. 1. With the exception of “research needs”, aspects relevant to each of these stages are subsequently discussed below. The discussion is grounded in reference to the recognition and description of potential uncertainty liable to manifest in in silico toxicology methods.

### Applying higher-level conceptual components for in silico toxicology methods

#### Problem framing

In the application of in silico toxicology methods, the framing of a problem begins when questions are raised about whether the methods are called for and, if yes, whether a selected method is suitable for a specific use case scenario, such as to predict an adverse effect that may be posed by a chemical of concern (particularly under plausible exposure scenarios) (Sauve-Ciencewicki et al. 2019).

As in silico methods are commonly used as data-filling techniques whose main purpose (e.g., during chemical risk assessment, classification, or prioritization) is to generate new or additional data for data-poor chemicals (Cronin and Madden 2010; Pastoor et al. 2014; Wang et al. 2012), problem framing may initially occur at the development stage of a chemical or drug compound, where screening is done to identify and eliminate potentially toxic properties. Alternatively, new or existing substances may be screened to determine whether or not a further risk assessment is required (Wadood et al. 2013).

#### Problem exploration

The second phase of the PF framework, problem exploration, includes the identification and organization of relevant knowledge and knowledge gaps, with the goal of developing a conceptual model and formulating a hypothesis. For in silico methods assessing the potential harm posed by chemicals such as cosmetic ingredients [e.g., coumarin (Baltazar et al. 2020)], exploring the problem (e.g., with regards to the potential of harm) could, for example, include looking beyond the concentration of the ingredient in a product and furthermore considering use-related factors such as frequency of use, inter-individual variations in use frequency and amount encountered per use. In addition, it would extend to consideration of potentially reactive metabolites (Baltazar et al. 2020). Taking these data into account, the problem exploration phase leads to a more in-depth evaluation of whether a specified model is adequate for addressing the intended prediction problem. Take a physiologically-based toxicokinetic (PBTK) model as an example. It is necessary for a modeler to explore whether the PBTK model is suitable to predict, for example, the dose of coumarin that is causally linked to a specific toxic response in a particular organ (e.g., human lungs and heart). This exploration may include asking (1) whether the model structure reflects and can incorporate chemical-specific information (plasma protein binding, blood partition coefficients, molecular weight, solubility, hydrophobicity, etc.) and physiological information (e.g., blood flow and organ volumes) necessary for the prediction; and (2) whether the model is adaptable to predict different exposure scenarios specific to coumarin (Baltazar et al. 2020). In addition, exploration will include considering how the acceptability of the PBTK prediction results might be evaluated or how the prediction results might fit into the overall weight of evidence decisions regarding the toxic effects of coumarin.

A lack of inclusion of particular information that might give deeper insight into a problem may introduce uncertainty in understanding such a problem, especially for complex problems (e.g., reproductive and developmental toxicity) whose accurate prediction depends on the levels of details (e.g., molecular descriptors and mechanistic characteristics) included in a model (Solomon et al. 2016). A study by Low et al. (2011) explains this point, where, the poor predictive performance of a QSAR model predicting hepatotoxicity of a collection of pharmaceuticals such as acetaminophen was attributed to the model’s failure to account for the influence of reactive metabolites (e.g., within acetaminophen). Accordingly, the authors suggested that such factors must be explored during the design of such QSAR model.

#### Conceptual model

Several scholars (Devos et al. 2019; Sauve-Ciencewicki et al. 2019; Solomon et al. 2016; Wolt et al. 2010) hold that a conceptual model should be iteratively developed during the framing and exploration phases (see Fig. 1). In theory, such a conceptual model should help in the development of testable hypotheses and operational strategies to enable data acquisition and the prioritization of information. This in turn leads to establishing the structural representation of an in silico model, which involves defining the model system boundary, variables, parameters and assumptions, and relationships among variables, between input and variables and between variables and output (Walker et al. 2003). Establishing the structural representation of an in silico model also includes clarifying the strengths and limits or suitability of the model for predicting a defined problem in a specific use case scenario (Walker et al. 2003). A conceptual model may also be held to be a useful tool when communicating the nature of a problem to stakeholders outside the PF team.

**Fig. 2:**
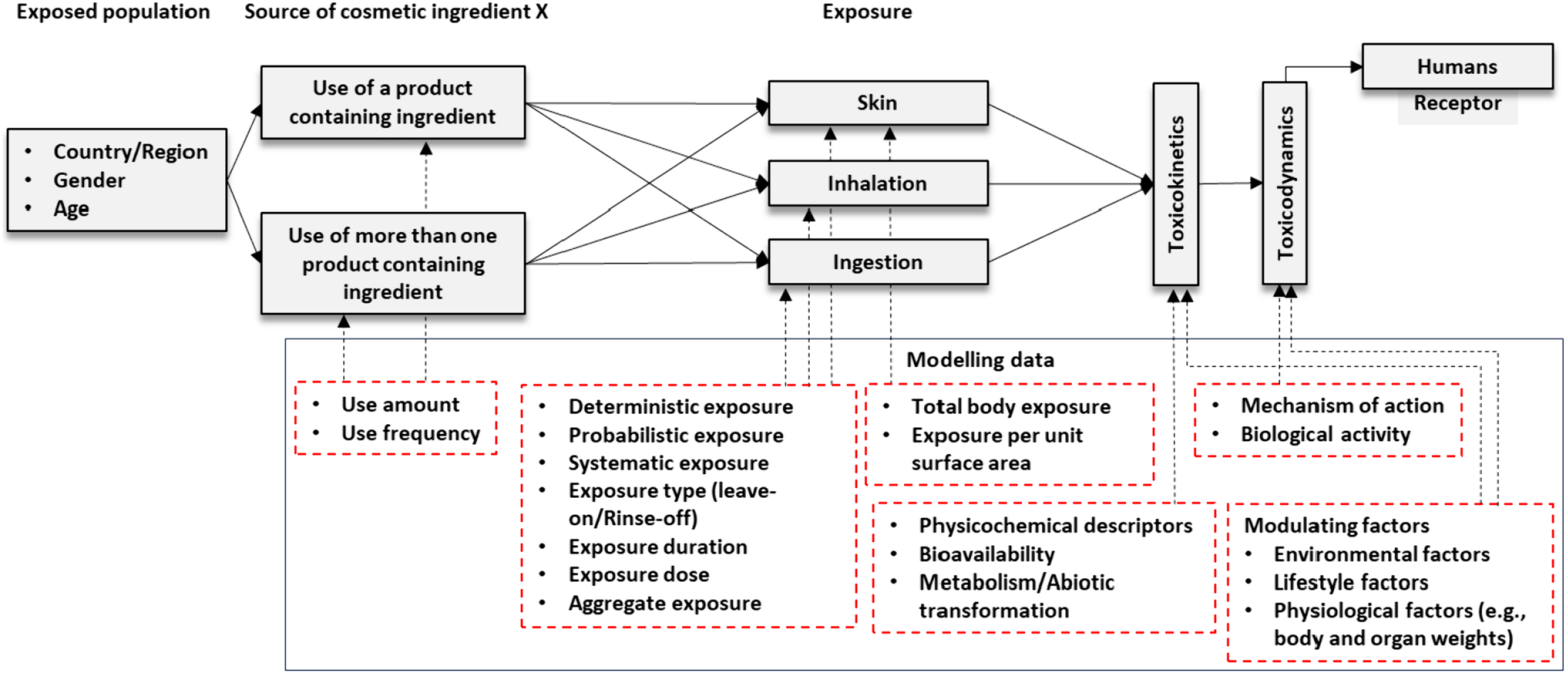
A simple conceptual model for risk assessment of a cosmetic ingredient X. The complete arrows show the assessment steps, while the dashed arrows show the possible in silico modelling data (in the lower box) required for each step

In practice, a conceptual model may take forms such as flow charts, simple statements, or diagrams (Wolt et al. 2010). For illustrative purposes only, we use a simple diagrammatic hypothetical conceptual model intended for a QSAR risk prediction of cosmetic ingredient X in humans (Fig. 2) to explain this. In silico methods like QSAR are particularly important in this illustration, as in the absence of experimental data, the methods are often applied to provide information on cosmetic ingredients through, for example, hazard identification (European Commission 2016).

In the case illustrated in Fig. 2, the starting point is to identify the target population being investigated, especially, when setting out to estimate realistic exposures based on, for example, inter-individual variability or frequency and amount of exposure on a population scale. The population can be identified by common characteristics, such as age, gender, consumers of a given country, or a population with a unique susceptibility to the chemical (Hall et al. 2007). The next step involves outlining possible sources of the ingredient (from shampoo, facial moisturizer, body lotion, etc.) that need to be considered. Ideally, a comprehensive QSAR prediction should consider all sources of the substance X (to facilitate aggregate exposure estimation using co-use scenarios), taking into account all exposure routes and potential effects on the exposed individuals. In practice, however, it is not uncommon for modelers to establish a boundary to limit the scope of the prediction to reduce the level of complexity of the model and its prediction, make simpler the interpretation of the model output, or take into account specific regulatory considerations (OECD 2018). The conceptual model is then expanded by adding all possible exposure pathway scenarios to simulate the exposure magnitude, which includes dermal, inhalation, and/or ingestion exposures. Upon exposure, toxicokinetic or toxicodynamic fates of the chemical are considered.

The next step in this conceptualization process (shown by the dashed arrows) is to identify the potential data elements (or parameter data) defining each entity being considered for the prediction of the toxicokinetic and/or toxicodynamic fates of the chemical X. The data should reflect, among others, the target endpoint, specific chemical exposure pathway(s), as relevant to each entity identified. In other words, to successfully use the QSAR model in this prediction context, a modeler needs to consider the relevance and reliability of the data, suitability of model structure with respect to the data or any specific prediction question asked (e.g., which exposure scenario and chemical mechanisms are being predicted?) and consider the sensitivity of the model to the anticipated input parameters.

In this context of QSAR, as explained by Cronin and Livingtstone (2004), the conceptual model in Fig. 2 is expected to describe the linkages between the independent variables (e.g., the ingredient's structure) and the dependent variables (e.g., toxic effect). An appropriate variable selection procedure should be followed to ensure the most appropriate variables that are statistically relevant in terms of the correlation between the variables are selected. It is not uncommon to use more than one variable (e.g., physiochemical descriptors for the ingredient X and, if applicable, for the ingredient’s metabolites) to predict the target biological activity. Cronin and Livingtstone (2004) emphasize that, in such cases, the QSAR model should specify all the variables considered and, if needed, indicate the expected order of influence on the expected biological activity. Translated to the exploration of a problem more generally in the context of in silico toxicology prediction, the exploration phase should lead to a conceptual model that describes plausible scenarios through which harm may arise from the chemical(s) that are being assessed—e.g., different exposure scenarios in the case illustrated in Fig. 2.

Notably, none of the in silico method-related studies identified in the Section “Formulating a general PF framework” include a conceptual model. Consequently, the authors do not discuss which variables might be included in such a model. We agree with Robinson et al. (2015), who argue that no quantitative model can exist without an underlying conception of its form. According to the authors, a lack of documentation of conceptual underpinnings makes it uncertain as to how to evaluate the completeness, clarity, and consistency of the logical structure behind the predictive tool derived. For in silico toxicological models, the lack of an expressed concept further makes it difficult to ascertain fitness for purpose based solely on the variables included.

#### Hypothesis formulation

An important role of the conceptual model is to function as a basis for the creation of testable hypotheses (Sauve-Ciencewicki et al. 2019). In the context of in silico models for chemical risk assessment, this amounts to generating a risk hypothesis based upon credible assumptions of how exposure to a chemical might affect a biological system. Consider the example of triethanolamine (a surfactant or stabilizer used in cosmetic ingredients)—a substance associated with incidences of liver tumors in animal studies (National Toxicology Program 2004). Consumers using triethanolamine-containing cosmetic products (e.g., moisturizers and facial cleansers) will experience systemic exposure to the compound following its dermal absorption (National Toxicology Program 2004). As such, it may be hypothesized that consumers regularly using the products will be at increased risk of developing carcinogenicity. As outlined in this example, the hypothesis is formulated using existing information both about exposure to and potential for triethanolamine to cause harm. Additionally, the hypothesis is based upon National Toxicology Program (2004) classification criteria of carcinogenicity of chemicals – i.e., a chemical is reasonably anticipated to be a human carcinogen based on evidence of carcinogenicity from animal studies, which indicates incidence of tumors at multiple tissue sites, by multiple exposure routes, etc.

**Fig. 3:**
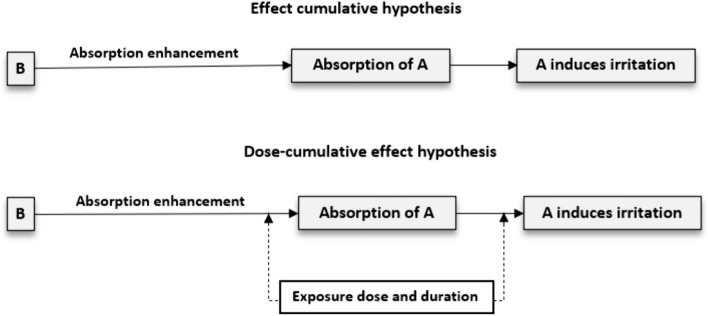
Simple hypotheses formulation diagrams showing two possible pathways to skin irritation: Effect-cumulative hypothesis (upper diagram) involving absorption enhancer B and skin irritant A, and dose-cumulative effect hypothesis (lower diagram) involving absorption enhancer B and irritant A and time and dose as the influencing factors

In the general PF literature, it is underlined that the development of a hypothesis is an iterative process, the outcome of which has the power to increase clarity and transparency in the defining and testing of postulated harm and, thus, increase confidence in the planned model prediction (Devos et al. 2019; Raybould 2006; Solomon et al. 2016; Wolt et al. 2010). The outcome of this process might also signal the need to revisit and adjust the earlier steps carried out in the PF process, either to match the hypothesis or to develop a new model. We illustrate this by drawing on a hypothetical QSAR model predicting the skin irritation potential of a low-dose mixture of two cosmetic ingredients: skin irritant (A) and skin absorption enhancer (B). Assume the original hypothesis is *B enhances skin absorption of skin-irritant A, which induces irritation*. Here, the hypothesis is formulated to only consider an effect-cumulative model, whereby the ingredient is held to produce a distinct influence (enhancing dermal absorption or inducing irritation)—the cumulative impact of which is skin irritation (Fig. 3). However, if the hypothesis is adjusted to include dose-cumulative effects of A, as follows: *the dose and duration of exposure to skin irritant A and ingredient B, which enhances skin absorption of A, determine the level of skin irritation*, it becomes necessary to revisit and reframe the problem to include both the dose and time factors of the ingredients (Fig. 3), as key QSAR parameter data.

As explained in the above example (Fig. 3), the QSAR prediction hypothesis should involve a comparison of possible hypotheses to ensure that necessary modelling data and model variables are incorporated into the model. To do this, one could first gather information about the product containing the target ingredients (in our case, A and B) to identify key properties and toxicological and toxicokinetic information about them (e.g., skin absorption and irritation potential), as these are crucial for designing the model. The idea is to formulate an initial hypothesis about the toxicological effects (and possibly underlying modes of action) and toxicokinetic fates of the ingredients, but also to formulate initial thoughts on whether the model would be fit for the intended prediction. Once done, the need for one, or more than one, hypothesis is evaluated; whereafter, based on the available supporting evidence, one can prioritize the hypothesis to explore based on the hypothesis: supported by the evidence, tenable but not well supported by the evidence, untenable, or unable to rule out.

Overall, similar to Wolt et al. (2010), we hold that rigorous, transparent and iterative examination and consideration of various possible hypotheses, as part of the PF process (e.g., effect-cumulative hypothesis and dose-cumulative effect hypothesis in reference to Fig. 3), is required to ensure confidence in the prediction either that harm will result via a particular pathway, or else that this is highly unlikely (thus ruling out the need for its further analysis).

### PF components in studies of in silico toxicology methods

Analyzing the 13 papers in the Section “Identifying PF components in the general risk assessment literature” led to the identification of 15 distinct components that are relatable to practices and features common to the application of predictive in silico toxicology (Table 1). In some cases, the component is explicitly mentioned, with the authors clearly describing the component in a specific assessment context, including giving detailed explanations through examples. For example, Pestana et al. (2021) explicitly mention “no observed adverse effect level (NOAEL)” for determining the risk of triazoles from oral 90-day studies in rats as the assessment endpoint. In other cases, the components are implicitly mentioned through a description in a general context without, for example, providing examples that explain it. For example, Escher et al. (2019), in a general context, mention “sub chronic” as the exposure duration without specifying (e.g., by giving examples) the exact duration of the assessment.

**Table 1:** Problem formulation components in thirteen in silico method-related studies that include problem formulation as part of the study

Component category	Specific component	References
Context of use	Prioritization, hazard screening, risk assessment/classification and labelling	Parish et al. (2020); Cronin et al. (2019a, b)
Assessment endpoint	Test species (rat)	Pestana et al. (2021)
	Hepatotoxicity, reproductive toxicity	Pradeep et al. (2020)
	NOAEL	Pestana et al. (2021)
	Reproductive endpoints	Ouedraogo et al. (2022)
	Benchmark values	Escher et al. (2019)
Exposure scenario	Exposure pathway (oral)	Escher et al. (2019); Pestana et al. (2021)
	Exposure pathway (dermal)	Ouedraogo et al. (2022); Reynolds et al. (2021); Baltazar et al. (2020)
	Exposure dose (0.1% face cream and 1% deodorant)	Reynolds et al. (2021); Baltazar et al. (2020)
	Exposure frequency	Sewell et al. (2017)
	Exposure duration (90-days and sub-chronic)	Escher et al. (2019); Pestana et al. (2021); Pradeep et al. (2020)
Decision context	Acceptable level of uncertainty	Dent et al. (2018); Belfield et al. (2021); Pallocca et al. (2022) Schultz et al. (2019); Escher et al. (2019)
	Allowable lifetime exposures	Escher et al. 2019)
	Acceptable safe concentrations	Ouedraogo et al. (2022)
	Acceptable hazard	Ouedraogo et al. (2022; Ball et al. (2022)

The identified components were used to formulate broader categories that cover both specific components/with examples (i.e., component categories)) based on the insights from the general PF literature. For example, the US EPA (US EPA 2016) describes “assessment endpoint” as "an explicit expression of the environmental value that is to be protected, operationally defined by an ecological entity and its attributes". Examples of assessment endpoints include a receptor of concern (e.g., test species) and the characteristics of the receptor to be measured (e.g., reproductive toxicity in the test species) (US EPA 2016). From our analysis of the specific components, test species, NOAEL (Pestana et al. 2021), hepatotoxicity and reproductive toxicity (Pradeep et al. 2020), reproductive endpoints (Ouedraogo et al. 2022), and benchmark values (Escher et al. 2019) fit the description of assessment endpoints; thus we used the “assessment endpoint” as the broader component category to represent these specific components/with examples (see Table 1 for the other formulated component categories).

Our analysis of the components across the 15 papers identified in the Section “Formulating a general PF framework” revealed little general consistency regarding the components addressed—with minimal overlap generally present. A majority of the components (8/15) were mentioned in only a single publication (e.g., frequency of exposure (Sewell et al. 2017)), whereas 4 were referenced in 3 or more papers [e.g., assessment endpoint Enoch 2010; Escher et al. 2019; Pestana et al. 2021; Pradeep et al. 2020)], and not one appeared within more than 5 publications. Furthermore, the form in which the components manifested varied considerably, with 7/15 explicitly mentioned and the rest implicitly mentioned. An example is Pestana et al. (2021) who explicitly mention NOAEL as the assessment endpoint, while Ouedraogo et al. (2022) adopt a more general category (i.e., reproductive endpoints). Although such characterization by Ouedraogo et al. (2022) can provide a general idea of the assessment endpoint targeted by developers and users of in silico toxicology models, we hold that a more specific characterization could provide added value by addressing uncertainty about which specific reproductive endpoint is targeted. Notably, components describing specific model features (i.e., those relating to exposure scenarios) were addressed with greater frequency than those covering conceptual aspects (i.e., associated with the context of use).

The lack of components related to higher-level concepts (e.g., conceptual model, problem framing, and hypothesis development) in the analyzed studies presents a gap in the in silico toxicology PF, as the need to incorporate these concepts within such a PF has been emphasized by several authors (Callahan and Sexton 2007; Devos et al. 2019; Nickson 2008; Paoli et al. 2022; Solomon et al. 2016).

### Uncertainties associated with the higher-level components of PF

By first analyzing and reflecting on the PF components identified in the 13 publications under section “Applying the PF framework to in silico toxicology methods”, we considered PF as a potential area where uncertainty can occur with respect to the higher-level components in instances where particular components are missing (thus leading to a partial inclusion of components) or only implicitly described (i.e., generality in the description of PF components such that a component does not provide any specificity in a given model prediction context). In this section, we discuss potential sources of uncertainty within PF components and propose a process that may be followed to characterize and address the uncertainty.

#### Sources of uncertainty

##### Problem framing

In the problem-framing process, uncertainty generally arises, especially as there are commonly different (and not seldom conflicting), yet legitimate and plausible, basis for concerns regarding a chemical. Additionally, uncertainty might arise where a problem is simplified to reduce complexity in its interpretation. In determining whether in silico methods are called for, it is necessary to ascertain if there is sufficient data or scientific rationale to support the evidence about the potential harm posed (Madden et al. 2020; OECD 2019). In cases where there is sufficient evidence, or an estimate of potential harm can be gained by other methods, in silico methods are generally not called for. Where in silico methods are called for, uncertainty still remain of whether an in silico model (e.g., read-across or QSAR) is robust or reliable for use (as a standalone or in an integrated system) for a particular toxicity prediction. Alternatively, uncertainty will arise from questions on whether a PF sufficiently describes the model to provide a starting point for judging the scientific validity of its predictions or acceptability of the prediction outcome (US EPA 2012). With respect to QSAR models, this may be identified through consideration of model form—i.e., is it quantitative (i.e., statistical regression), or qualitative (such as read-across or structural alert-based model) (US EPA 2012)? The knowledge drawn from answers to such a question could offer meaningful insights into the strength of the models or the extent to which the models can be applied for certain applications (e.g., hazard identification, or risk assessment) (Parish et al. 2020). The knowledge could also be drawn upon to facilitate the framing of the level of importance of model features (model algorithms, descriptors, etc.) for the defined model use case scenario.

It is important to recognize that each framing will lead to the inclusion and exclusion of different aspects of the broader problem, depending on the viewpoints or assumptions considered at the PF stage. This in turn, sets the model system boundaries drawn for its assessment, such as the model structure (e.g., variables and variables relationships) (Sluijs et al. 2008). Articulation of the problem framing and related concerns of an in silico model is a process designed to facilitate a common understanding of the utility context of the model and its predictions. If the problem is not clearly framed, it might remain uncertain what aspects that are salient to influence the choice of a model (e.g., its applicability domain, parameters and fitness evaluation criteria)—and what knowledge is relevant in prediction and evaluation (Sluijs et al. 2008). Ideally, the outcome of the problem-framing process is a statement that leads to describing information and research needs (Devos et al. 2019; Sauve-Ciencewicki et al. 2019; Solomon et al. 2016).

##### Problem exploration

Overall, as with problem framing, a clearly explored problem should minimize the manifestation of uncertainty by unambiguously describing the hazards or risks associated with a chemical and including the necessary details in the proposed model to help in gaining a deeper understanding of its suitability to make the prediction. This point can be illustrated by referring to the study by Moss et al. (2016) on skin sensitization of cinnamyl alcohol. The authors acknowledge the common understanding that cinnamyl alcohol is a pre-hapten whose skin sensitization can occur through conversion to protein-reactive cinnamaldehyde. However, their further exploration reveals that cinnamyl alcohol can also directly induce skin sensitization through a pathway independent of the one involving cinnamaldehyde. This conclusion was supported by observation of the formation of epoxy-alcohol and the activation of the allylic hydroxyl function. Here, uncertainty can be introduced if cinnamaldehyde data, information on possible additive/synergistic reaction of cinnamyl alcohol and cinnamaldehyde, or the influence of exposure dose and duration of each compound, etc., are not considered in a model prediction. In other words, as shown in this example and emphasized elsewhere (Sauve-Ciencewicki et al. 2019), it is possible to gain a more comprehensive understanding of a problem by organizing relevant knowledge about the chemical for in silico model development and prediction and ensure a well-defined applicability domain and adequacy of the model to address the prediction problem; otherwise, the model may suffer from inadequacy in terms of the input parameters and model structure used. As the central goal for the problem exploration step is to lead to the development of a conceptual model, the quality of the conceptual model will inevitably suffer given to the uncertainties in problem exploration (Devos et al. 2019; Sauve-Ciencewicki et al. 2019).

##### Conceptual model

El-Ghonemy et al. (2005) as well as Zheng and Bennett (2002) emphasize the need to pay attention to possible uncertainty arising from either the under-simplification or oversimplification of a conceptualized model. Generally speaking, an oversimplified conceptual form fails to capture the crucial features necessary for the successful construction of a quantitative model, thus resulting in a model that inadequately simulates the endpoint intended. Ekins et al. (2007) illustrate this uncertainty by describing two chemical interaction systems: toxicodynamic and toxicokinetic. The former should incorporate reference to toxic responses of the biological system after chemical exposure; thus, the conceptual model should capture those elements of the biology (e.g., receptors, ion channels, nucleic acids, anabolic and catabolic enzymes) implicated in the emergence of toxicity. In the latter case, the conceptual underpinning should capture those elements of the physiological response to the xenobiotic presence (e.g., chemical-metabolizing enzymes, transporters, circulating proteins) that serve to elicit or influence either the metabolism, transportation, distribution or excretion of the chemical (Ekins et al. 2007). In these instances, oversimplification of the conceptual model might occur if, for example, the number of biological elements included is reduced to the point where connections between themselves and other variables are not captured. In contrast, under-simplification will generally introduce several variables into a model system, without clearly distinguishing between them. Thus, uncertainty is introduced due to the resultant difficulties in interpreting the influence of any single feature upon the output of the model.

The above discussions highlight the importance of developing and using a conceptual model to clarify the boundaries of an in silico model system, as well as the variables and relationships which are conceived as relevant to it. In turn, a conceptual model will help to establish whether it is appropriate to include or exclude specific information in the quantitative model and, thus, by extension, to infer the utility of its predictions (Walker et al. 2003). This implies that it is crucial to make explicit the model boundaries, as a lack of clarity on this matter will introduce uncertainty regarding which variables are appropriate to include or exclude (Skinner et al. 2014). Furthermore, confidence in a developed quantitative approach and its predictive output will require documenting any simplifications, assumptions, and justifications provided for the choice of information considered within the conceptual model.

##### Hypothesis formulation

A hypothesis formulation should account for possible associated uncertainties. In reference to the example in Fig. 3, the choice of any hypothesis should clearly include the understanding of the expected level of uncertainty surrounding the exposure pathway selected and how that could translate to the level of confidence placed on the predicted skin irritation. If, for example, uncertainty is expected to be “too high”, then decision could be iteratively made to add information (e.g., information on the exposure dose/duration) to lower the uncertainty.

Welss et al. (2004) present explanations that corroborate the above discussion on uncertainty. The authors point out that substances can operate through two distinct pathways to initiate skin irritation. In the first, damage to the barrier function of the stratum corneum can initiate irritation, with the ingredients’ dose and duration of exposure determining the extent of any injury (Johnson et al. 2020; Welss et al. 2004). The second pathway occurs when damage to the skin enhances irritants' penetration into the deeper epidermal layers, initiating irritation through interaction with living keratinocytes (Johnson et al. 2020; Welss et al. 2004). Both routes can lead to skin irritation, whether alone or in combination. The hypotheses may be constructed based upon each of these putative pathways (pathway 1 or pathway 2)—alongside a third, combining elements of both pathway 1 and pathway 2. Uncertainty might otherwise remain in relation to factors such as the scenario anticipated in the context of the prediction, the level of confidence which should be held in the scenario chosen, the number and type of parameters which ought to be included in the model, and the robustness of the prediction output. Overall, we contend that developing and evaluating several hypotheses makes it easier to not only judge whether or not any one selected is robust but also whether it is fit for a specified hazard or risk prediction. Alternatively, the uncertainty associated with hypotheses formulation might set a precondition for rejecting in silico predictions for use in a regulatory setting.

#### Characterizing and addressing uncertainty associated with PF components

**Fig. 4:**
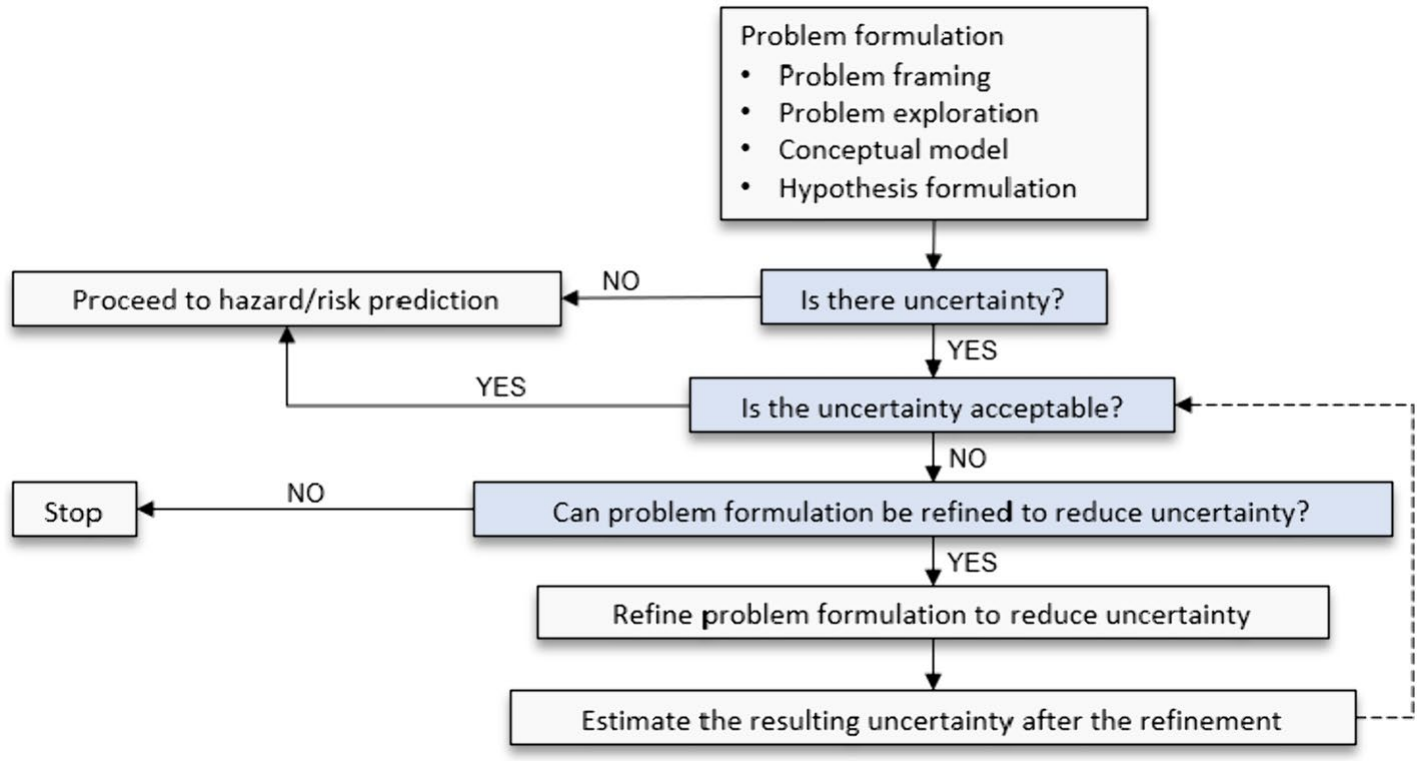
A proposed step-step (shown with the complete arrows) process to characterize and address the uncertainty associated with PF components and an iteration (shown with the dashed arrow) required at one of the steps

Following on from the discussion under Section “Sources of uncertainty” above, we propose a process (Fig. 4) that one can follow to characterize and address the uncertainties. Description of the PF components (and identification of areas of uncertainty) is a critical first step in this process, as it provides the context for uncertainty analysis. Three fundamental questions (shown in the light blue boxes in Fig. 4) are then raised. The first question (“Is there uncertainty?”) seeks to determine whether uncertainty resides within any of the described components. In theory, a “no” answer can emerge, indicating that uncertainty is not a concern; thus ruling out the need for uncertainty analysis but indicating the need to directly proceed to predict chemical hazard/risk following problem formulation. If, however, the answer is “yes”, then the second question (“Is the uncertainty acceptable?”) becomes relevant to ask in a defined decision context.

To determine the acceptability of uncertainty in PF, it is necessary to first characterize (i.e., quantify/qualify) uncertainty from each component—this can be done, for example, using a scoring scheme, such as “low” or “high” to rank uncertainty. Here, the goal is to determine how each component contributes to the overall uncertainty in PF as well as prioritize areas for uncertainty reduction (or elimination, if possible). It is necessary to clearly spell out the context in which the level of acceptability is defined, as different uncertainty levels might be considered as being acceptable depending upon the potential consequences of inaccurate formulations. For example, a high uncertainty level can be accepted for decisions with respect to priority-setting—e.g., a machine learning-based model for screening chemical inventories to identify toxic molecules can be acceptable even with formulation that leads to relatively high false positives in the prediction, as long as the model is fit for the screening purpose (Belfield et al. 2021). On the other hand, low uncertainty in a mechanistic-based model risk prediction might be accepted when setting health-based standards (Belfield et al. 2021). Overall, if the level of uncertainty is considered acceptable, one can proceed to the hazard/risk prediction phase; otherwise, an additional question should be raised about whether PF can be refined to reduce the uncertainty to an acceptable level.

When uncertainty is considered unacceptable and irreducible, one may choose to stop the need for analysis and potentially consider not using the PF. However, if uncertainty can be reduced, one can evaluate the added value of refining the components associated with uncertainty by, for example, incorporating more information or introducing more complex models. After the refinement, the resulting uncertainty can be estimated to determine whether or not the uncertainty has been reduced to an acceptable level. The essence of this step is to allow this process to be iterative by making it possible to continuously identify, estimate, and address uncertainty within PF components for a specific prediction context.

### Further consideration of PFs

Finally, we wish to highlight an area where future research is sorely needed, Sauve-Ciencewicki et al. (2019) emphasize that the PF process must be structured, iterative, and include *all key stakeholders*, as it is context-dependent (our emphasis). We agree that it is crucial to recognize that each problem situation is unique, and that the quality of the decision outcome depends on the perspectives and expertise of those included in the process. However, including *all* key stakeholders, including relevant experts, as proposed by the authors, is a tall task. Also, the framework proposed by the authors assumes that the deliberations will generate a consensus and that “Failure to reach consensus on the specific problem to be addressed leads to misunderstandings and an inability to create appropriate solutions" (ibid., p. 187). We argue that this is a weakness in the PF frameworks we have reviewed, as it is well documented that in cases of societal relevance where there is large uncertainty, it is generally the case that people—including the experts—do not reach a consensus, and that more research and deliberations can lead to hardened positions (Donfrancesco et al. 2023; Funtowicz and Ravetz 1993; McIlroy-Young et al. 2021). Notably, little, if anything, is known about how different producers or users of in silico model predictions prioritize among different PF components or sources of uncertainty.

## Conclusion

Our study led to the discovery of a gap between the broader risk assessment literature and in silico toxicology method literature about how PF is conceptualized. While the general PF literature emphasises that PF frameworks must address higher-level conceptual and context-specific questions, the studies we analyzed, which all describe PFs for in silico toxicology methods, do not include such components. Furthermore, there was very little consistency across the studies regarding the type of components they addressed. Drawing on a general PF framework (Sauve-Ciencewicki et al. 2019), we developed a preliminary PF framework (Fig. 1) for in silico toxicology methods and described the framework in light of the components mentioned in studies that address PFs applied to in silico toxicology methods (Table 1). To our knowledge, such a framework has not been previously developed for this purpose. The framework can be used to clarify which PF components are central to a particular in silico toxicology method. Critical to this is to shed light on how uncertainty can manifest within the PF, if particular components are excluded or implicitly described. Our study suggests that explicit-making the selection among components has the potential to clarify the perspectives used in the selection process and help avoid potential biases and blind spots in the team that developed the PF. For a growing research field such as in silico toxicology methods, where scholars from different disciplinary and cultural backgrounds are likely to differ in their prioritization of which components to include in a PF, chemical regulatory decisions are likely to benefit from improved transparency to that end (Devos et al. 2019).

### Supplementary Information

Below is the link to the electronic supplementary material.

Supplementary file1 (DOCX 60 KB)
