# Supplementary material for: A problem formulation framework for the application of in silico toxicology methods in chemical risk assessment
Source: Arch Toxicol. 2024 Mar 30;98(6):1727–40. doi: 10.1007/s00204-024-03721-6 (PMC11106140; doi:10.1007/s00204-024-03721-6)
Supplement: Supplementary file 1 — Supplementary file1 (DOCX 60 KB) [file 204_2024_3721_MOESM1_ESM.docx]

**Supplementary Material**

**A Problem Formulation Framework for the Application of *In Silico* Toxicology Methods in Chemical Risk Assessment**

Jerry Achar^1^, Mark T.D. Cronin^2^*, James W. Firman^2^, Gunilla Öberg^1^

^1^Institute for Resources Environment, and Sustainability, The University of British Columbia, 2202 Main Mall, Vancouver, BC V6T 1Z4, Canada

^2^School of Pharmacy and Biomolecular Sciences, Liverpool John Moores University, Byrom Street, Liverpool, L3 3AF, UK

*Corresponding author:

E-mail: M.T.Cronin@ljmu.ac.uk (Mark T.D. Cronin)

Table S1. List of studies in the general risk assessment literature (outside of *in silico* methods or alternative to animal testing approaches in general) describing higher-level conceptual problem formulation components.

| **Author** | **Higher-level component** |
| --- | --- |
| Bette at al. (2013) | Assessment context (prioritization and screening); Endpoint; Safe dose; |
| Callahan and Sexton (2007) | Conceptual model; Analysis plan |
| Jones and Gallek (2004) | Assessment objectives; Spatial and temporal scales; Measurement endpoints |
| Nickson (2008) | Assessment Endpoints; Conceptual Model; Analysis Plan |
| Poli et al. (2022) | Assessment question (scientific questions to be addressed); Conceptual model; Risk hypothesis; Legislative context an assessment; Research needs |
| Embry et al. (2014) | Scenario description (e.g., chemical use); Highlighting existing knowledge; Context description (e.g., potential exposure scenario); Defining acceptable margin of exposure; Regulatory options |
| Devos et al. (2019) | Conceptual model; Formulating hypothesis; Defining what qualifies as harm |
| Felter et al. (2021) | Problem scoping; Setting up hazard or risk analysis plan; Purpose of the assessment (hazard identification and classification or risk assessment) |
| OECD (2019) | Assessment scope and goals; Acceptable level of uncertainty; Defining urgency of the assessment |
| Pastoor et al. (2014) | Defining chemical exposure |
| Sauve-Ciencewicki et al. (2019) | Framing problem; Framing problem; Problem Statement; Conceptual Model |
| Solomon et al. (2016) | Planning and scoping; Identifying and characterizing stressors; Conceptual Model; Plan of Analysis |
| USEPA (2016) | Stressors (duration of persistence and frequency of occurrence); Sources (e.g., background levels), exposure (media and routes); Susceptibility and sensitivity of the receptor |
| Wolt et al. (2010) | Assessment endpoints; Risk hypotheses; Conceptual model; Exposure; Level of Uncertainty |
| World Health Organization/International Programme on Chemical Safety (2018) | Acceptable levels of uncertainty and risk; Assessment endpoints; Exposure scenarios; Analysis plan and information needs; Risk management scope and assessment goals |

Table S2: List of the thirteen in silico method-related published in the peer-reviewed papers that were analyzed in the present study. They were selected as they proposed or discussed the need for problem formulation in the development of in silico methods.

| **Author** | **Description of the study** |
| --- | --- |
|  |  |
| Parish et al. (2020) | Develop a framework for fit-for-purpose evaluation of NAM's application in the regulatory context of chemical risk assessment. The proposed PF calls for the specification of the regulatory use of NAM (i.e., chemical prioritization, hazard identification, or/and risk assessment). |
| Pestana et al. (2021) | Appraise uncertainty in read-across using Assessment Elements (AEs) from the European Chemicals Agency's Read-Across Assessment Framework (RAAF). A 90-day oral sub-chronic toxicity of triazole in rats was used as a case study. |
| Escher et al. (2019) | Outline a general read-across assessment to support hazard characterization of grouped compounds by generating data on the chemicals' dynamic and kinetic properties. |
| Baltazar et al. (2020), Reynolds et al. (2021) | Systematic toxicity assessment of 0.1% coumarin in face cream and body lotion in an exposure-led approach and a battery of NAMs (including *in vitro* assays, physiologically based kinetic models, and Skin Allergy Risk Assessment Model). |
| Ouedraogo et al. (2022) | Develop an illustrative 10-step read-across framework for propylparaben cosmetic safety assessment as a proof-of-concept for the value added by NAMs in next-generation risk assessment of chemicals. |
| Dent et al. (2018) | Propose principles for incorporating NAMs into risk assessments of cosmetic ingredients using the guidelines of next-generation risk assessment, such as ensuring assessments are human-relevant, exposure-led, hypothesis-driven and designed to prevent harm. |
| Belfield et al. (2021) | Evaluate quantitative structure-activity relationships models in terms of their uncertainty, variability and potential areas of bias by mapping out the models' components onto specific regulatory uses (i.e., risk assessment, classification and labelling, and screening and prioritization). |
| Cronin et al. (2019) | Identify opportunities and challenges to implementing *in silico* methods (e.g., quantitative structure-activity relationships and read across) to assess the safety of chemicals like pharmaceuticals, personal care products, and industrial chemicals. |
| Sewell et al. (2017) | Review the rate of progress in regulatory acceptance of non-animal methodology into regulatory and identify ways to expedite progress |
| Schultz et al. (2019) | Identify major sources of uncertainty (e.g., regulatory use of the prediction and data for the apical endpoint being assessed) that has the potential to impact acceptance of read-across argument. |
| Ball et al. (2022) | Develop a framework that incorporates *in silico, in vitro* and *in vivo* methods for REACH requirements in assessing chemical hazard and exposure using a tiered approach. |
| Pallocca et al. (2022) | Delineate how the practical applicability of NAMs and strategies will be deployed to establish an overall next generation risk assessment framework for chemicals and other substances like drugs. |

**References**

Ball, N., Bars, R., Botham, P. A., Cuciureanu, A., Cronin, M. T. D., Doe, J. E., Dudzina, T., Gant, T. W., Leist, M., & van Ravenzwaay, B. (2022) A framework for chemical safety assessment incorporating new approach methodologies within REACH. Arch Toxicol 96: 743–766. https://doi.org/10.1007/s00204-021-03215-9

Baltazar, M. T., Cable, S., Carmichael, P. L., Cubberley, R., Cull, T., Delagrange, M., Dent, M. P., Hatherell, S., Houghton, J., Kukic, P., Li, H., Lee, M.-Y., Malcomber, S., Middleton, A. M., Moxon, T. E., Nathanail, A. V., Nicol, B., Pendlington, R., Reynolds, G., … Westmoreland, C. (2020) A next-generation risk assessment case study for coumarin in cosmetic products. Toxicol Sci 176: 236–252. https://doi.org/10.1093/toxsci/kfaa048

Belfield, S. J., Enoch, S. J., Firman, J. W., Madden, J. C., Schultz, T. W., & Cronin, M. T. D. (2021) Determination of “fitness-for-purpose” of quantitative structure-activity relationship (QSAR) models to predict (eco-)toxicological endpoints for regulatory use. Reg Toxicol Pharmacol 123: 104956. https://doi.org/10.1016/j.yrtph.2021.104956

Callahan, M. A., & Sexton, K. (2007) If cumulative risk assessment is the answer, what is the question? Environ Health Persp 115: 799–806. https://doi.org/10.1289/ehp.9330

Cronin, M. T. D., Madden, J. C., Yang, C., & Worth, A. P. (2019) Unlocking the potential of *in silico* chemical safety assessment – A report on a cross-sector symposium on current opportunities and future challenges. Comput Toxicol 10: 38–43. https://doi.org/10.1016/j.comtox.2018.12.006

Dent, M., Amaral, R. T., Da Silva, P. A., Ansell, J., Boisleve, F., Hatao, M., Hirose, A., Kasai, Y., Kern, P., Kreiling, R., Milstein, S., Montemayor, B., Oliveira, J., Richarz, A., Taalman, R., Vaillancourt, E., Verma, R., Posada, N. V. O. C., Weiss, C., & Kojima, H. (2018) Principles underpinning the use of new methodologies in the risk assessment of cosmetic ingredients. Comput Toxicol 7: 20–26. https://doi.org/10.1016/j.comtox.2018.06.001

Devos, Y., Craig, W., Devlin, R. H., Ippolito, A., Leggatt, R. A., Romeis, J., Shaw, R., Svendsen, C., & Topping, C. J. (2019) Using problem formulation for fit-for-purpose pre-market environmental risk assessments of regulated stressors. EFSA Journal 17(S1): e170708. https://doi.org/10.2903/j.efsa.2019.e170708

Embry, M. R., Bachman, A. N., Bell, D. R., Boobis, A. R., Cohen, S. M., Dellarco, M., Dewhurst, I. C., Doerrer, N. G., Hines, R. N., Moretto, A., Pastoor, T. P., Phillips, R. D., Rowlands, J. C., Tanir, J. Y., Wolf, D. C., & Doe, J. E. (2014) Risk assessment in the 21st century: Roadmap and matrix. Crit Rev Toxicol 44(sup3): 6–16. https://doi.org/10.3109/10408444.2014.931924

Escher, S. E., Kamp, H., Bennekou, S. H., Bitsch, A., Fisher, C., Graepel, R., Hengstler, J. G., Herzler, M., Knight, D., Leist, M., Norinder, U., Ouédraogo, G., Pastor, M., Stuard, S., White, A., Zdrazil, B., van de Water, B., & Kroese, D. (2019) Towards grouping concepts based on new approach methodologies in chemical hazard assessment: The read-across approach of the EU-ToxRisk project. Arch Toxicol 93: 3643–3667. https://doi.org/10.1007/s00204-019-02591-7

Felter, S. P., Bhat, V. S., Botham, P. A., Bussard, D. A., Casey, W., Hayes, A. W., Hilton, G. M., Magurany, K. A., Sauer, U. G., & Ohanian, E. V. (2021) Assessing chemical carcinogenicity: Hazard identification, classification, and risk assessment. Insight from a Toxicology Forum state-of-the-science workshop. Crit Rev Toxicol 51: 653–694. https://doi.org/10.1080/10408444.2021.2003295

Jones, C., & Gilek, M. (2004) Overview of programmes for the assessment of risks to the environment from ionising radiation and hazardous chemicals. J Radiol Prot 24(4A): A157–A177. https://doi.org/10.1088/0952-4746/24/4A/010

Meek ME, B., Bolger, M., Bus, J. S., Christopher, J., Conolly, R. B., Lewis, R. J., Paolini, G. M., Schoeny, R., Haber, L. T., Rosenstein, A. B., & Dourson, M. L. (2013) A framework for fit-for-purpose dose response assessment. Reg Toxicol Pharmacol 66: 234–240. https://doi.org/10.1016/j.yrtph.2013.03.012

Nickson, T. E. (2008) Planning environmental risk assessment for genetically modified crops: Problem formulation for stress-tolerant crops. Plant Physiol 147: 494–502. https://doi.org/10.1104/pp.108.118422

OECD (2019). Guiding Principles and key elements for establishing a weight of evidence for chemical assessment. OECD. https://doi.org/10.1787/f11597f6-en

Ouedraogo, G., Alexander-White, C., Bury, D., Clewell, H. J., Cronin, M., Cull, T., Dent, M., Desprez, B., Detroyer, A., Ellison, C., Giammanco, S., Hack, E., Hewitt, N. J., Kenna, G., Klaric, M., Kreiling, R., Lester, C., Mahony, C., Mombelli, E., … Cosmetics Europe.(2022) Read-across and new approach methodologies applied in a 10-step framework for cosmetics safety assessment – A case study with parabens. Reg Toxicol Pharmacol 132: 105161. https://doi.org/10.1016/j.yrtph.2022.105161

Pallocca, G., Moné, M. J., Kamp, H., Luijten, M., Water, B. van de, & Leist, M. (2022) Next-generation risk assessment of chemicals – Rolling out a human-centric testing strategy to drive 3R implementation: The RISK-HUNT3R project perspective. ALTEX - Altern Anim Ex 39(3): Article 3. https://doi.org/10.14573/altex.2204051

Paoli, G., Momoli, F., Tyshenko, M. G., Meek, M. E. B., & Krewski, D. (2022) Problem formulation for EFSA scientific assessments. EFSA Supporting Publications 19(7): 7349E. https://doi.org/10.2903/sp.efsa.2022.EN-7349

Parish, S. T., Aschner, M., Casey, W., Corvaro, M., Embry, M. R., Fitzpatrick, S., Kidd, D., Kleinstreuer, N. C., Lima, B. S., Settivari, R. S., Wolf, D. C., Yamazaki, D., & Boobis, A. (2020) An evaluation framework for new approach methodologies (NAMs) for human health safety assessment. Reg Toxicol Pharmacol 112: 104592. https://doi.org/10.1016/j.yrtph.2020.104592

Pastoor, T. P., Bachman, A. N., Bell, D. R., Cohen, S. M., Dellarco, M., Dewhurst, I. C., Doe, J. E., Doerrer, N. G., Embry, M. R., Hines, R. N., Moretto, A., Phillips, R. D., Rowlands, J. C., Tanir, J. Y., Wolf, D. C., & Boobis, A. R. (2014) A 21st century roadmap for human health risk assessment. Crit Rev Toxicol 44(sup3): 1–5. https://doi.org/10.3109/10408444.2014.931923

Pestana, C. B., Firman, J. W., & Cronin, M. T. D. (2021) Incorporating lines of evidence from New Approach Methodologies (NAMs) to reduce uncertainties in a category based read-across: A case study for repeated dose toxicity. Reg Toxicol Pharmacol 120: 104855. https://doi.org/10.1016/j.yrtph.2020.104855

Reynolds, G., Reynolds, J., Gilmour, N., Cubberley, R., Spriggs, S., Aptula, A., Przybylak, K., Windebank, S., Maxwell, G., & Baltazar, M. T. (2021) A hypothetical skin sensitisation next generation risk assessment for coumarin in cosmetic products. Reg Toxicol Pharmacol 127: 105075. https://doi.org/10.1016/j.yrtph.2021.105075

Sauve-Ciencewicki, A., Davis, K. P., McDonald, J., Ramanarayanan, T., Raybould, A., Wolf, D. C., & Valenti, T. (2019) A simple problem formulation framework to create the right solution to the right problem. Reg Toxicol Pharmacol 101: 187–193. https://doi.org/10.1016/j.yrtph.2018.11.015

Schultz, T. W., Richarz, A.-N., & Cronin, M. T. D. (2019) Assessing uncertainty in read-across: Questions to evaluate toxicity predictions based on knowledge gained from case studies. Comput Toxicol 9: 1–11. https://doi.org/10.1016/j.comtox.2018.10.003

Sewell, F., Doe, J., Gellatly, N., Ragan, I., & Burden, N. (2017) Steps towards the international regulatory acceptance of non-animal methodology in safety assessment. Reg Toxicol Pharmacol 89: 50–56. https://doi.org/10.1016/j.yrtph.2017.07.001

Solomon, K. R., Wilks, M. F., Bachman, A., Boobis, A., Moretto, A., Pastoor, T. P., Phillips, R., & Embry, M. R. (2016) Problem formulation for risk assessment of combined exposures to chemicals and other stressors in humans. Crit Rev Toxicol 46: 835–844. https://doi.org/10.1080/10408444.2016.1211617

US EPA. (2016). Phases of ERA - planning and problem formulation [Collections and Lists]. https://www.epa.gov/ecobox/phases-era-planning-and-problem-formulation. Accessed 14 March 2022

Wolt, J. D., Keese, P., Raybould, A., Fitzpatrick, J. W., Burachik, M., Gray, A., Olin, S. S., Schiemann, J., Sears, M., & Wu, F. (2010) Problem formulation in the environmental risk assessment for genetically modified plants. Transgenic Res 19: 425–436. https://doi.org/10.1007/s11248-009-9321-9

World Health Organization/International Programme on Chemical Safety (2018) Guidance document on evaluating and expressing uncertainty in hazard characterization. World Health Organization. https://apps.who.int/iris/handle/10665/259858
